# Intracellular pH-responsive and rituximab-conjugated mesoporous silica nanoparticles for targeted drug delivery to lymphoma B cells

**DOI:** 10.1186/s13046-017-0492-6

**Published:** 2017-02-06

**Authors:** Shoubing Zhou, Dan Wu, Xiaodong Yin, Xiaoxiao Jin, Xiu Zhang, Shiya Zheng, Cailian Wang, Yanwen Liu

**Affiliations:** 1 Department of Oncology, Zhongda Hospital, Medical School of Southeast University, Dingjiaqiao Road 87th, Nanjing, 210009 Jiangsu China; 2 Department of Oncology, The People’s Hospital, Jiangyin, Wuxi, 214000 Jiangsu China; 3 Department of Oncology, The People’s Hospital, Binhai, Yancheng, 224000 Jiangsu China

**Keywords:** Mesoporous silica nanoparticles, pH-response, Targeted drug delivery, B cell lymphoma, Rituximab

## Abstract

**Background:**

One of the main problems in B cell lymphoma treatment is severe adverse effects and low therapeutic efficacy resulting from systemic chemotherapy. A pH-sensitive controlled drug release system based on mesoporous silica nanoparticles was constructed for targeted drug delivery to tumor cells to reduce systemic toxicity and improve the therapeutic efficacy.

**Methods:**

In this study, the doxorubicin (DOX) was filled into the mesopores of the functional MSNs (DMSNs). Furthermore, rituximab was introduced as the targeted motif of functional DMSNs using an avidin-biotin bridging method to evaluate the targetability to tumor cells. Then, the cell viability and apoptosis efficiency after treatment with rituximab-conjugated DMSNs (RDMSNs) were estimated by using CCK-8 assay and flow cytometry, respectively. Additionally, the research in vivo was performed to evaluate the enhanced antitumor efficacy and the minimal toxic side effects of RDMSNs. Also, TUNEL staining assay was employed to explore the mechanism of antitumor effects of RDMSNs.

**Results:**

This targeted drug delivery system exhibited low premature drug release at a physiological pH and efficient pH-responsive intracellular release under weakly acidic conditions. The in vitro tests confirmed that targeted RDMSNs could selectively adhere to the surface of lymphoma B cells via specific binding with the CD20 antigen and be internalized into CD20 positive Raji cells but few CD20 negative Jurkat cells, which leads to increased cytotoxicity and apoptosis of the DOX in Raji cells due to the release of the entrapped DOX with high efficiency in the slightly acidic intracellular microenvironment. Furthermore, the in vivo investigations confirmed that RDMSNs could efficiently deliver DOX to lymphoma B cells by pH stimuli, thus inducing cell apoptosis and inhibiting tumor growth, while with minimal toxic side effects.

**Conclusions:**

This targeted and pH-sensitive controlled drug delivery system has the potential for promising application to enhance the therapeutic index and reduce the side effects of B cell lymphoma therapy.

## Background

Lymphoma remains one of the primary causes of cancer mortality globally. Systemic chemotherapy is an indispensable treatment for many types of lymphoma. Rituximab, a chimeric monoclonal antibody that can specifically interact with the CD20 antigen [1], combined with doxorubicin (DOX) have been extensively used for improving the prognosis of B cell lymphoma over the past few decades [2]. Although these treatments have favorable therapeutic effects in most cases, significant adverse effects may occur due to premature drug release prior to reaching the targeted sites and nonspecific biodistribution in normal tissues [3]. Therefore, exploring new therapeutic strategies is necessary for preventing drugs from prematurely releasing during blood circulation, controlling drug nonspecific biodistribution, reducing off-target toxicity, and improving the therapeutic efficacy.

Nanoparticles have been reported to serve as drug delivery carriers in the nanomedicine field. Recently, mesoporous silica nanoparticles (MSNs) have attracted much attention due to their favorable properties including good biocompatibility, high stability, tunable pore diameter, unique porous structure, large loading capacity, and easy surface functionalization [4–9]. The ordered pore network of these MSNs can effectively load drug within the channels. Additionally, MSNs can also be designed to trigger the release of a loaded drug in response to either external or internal stimuli, such as temperature [10, 11], light [12, 13], redox reactions [14–16], enzymes [17, 18], pH value [19, 20], and biomolecules [21, 22]. Among these stimuli, pH-responsive drug release mechanisms are considered as a facile and convenient method for controlled drug release using a low pH signal, because endosomes and lysosomes formed in cells after internalization of drug delivery carriers are relatively more acidic with a pH of 4.5–5.5 [23]. Therefore, various types of pH-responsive drug delivery carriers based on MSNs have been developed to control drug release via a pH signal in endosomes and lysosomes. However, the use of only single pH-responsive MSNs that are based on passive targetability via enhanced permeability and retention effects for drug delivery results in facile internalization by normal cells through an unspecific uptake method. Therefore, several studies have reported that the external surface of MSNs can be modified with tumor-specific ligands to improve the active targetability by increasing the affinity between the receptor and the ligand. Various types of targeted ligands, such as folate [24], peptides [25], glycyrrhetinic acid [26], hyaluronic acid [27], mannose [28], arginine-glycine-aspartate [29], DNA aptamer [30], and lactobionic acid [31], have been successfully conjugated to drug delivery carriers, leading to an enhanced anticancer drug therapeutic index. At present, these ligands primarily targeted solid tumors rather than lymphoma and were rarely used in clinical applications for cancer molecular targeting therapy due to unwanted immunogenicity. Therefore, the development of intracellular pH-responsive and active targeted drug-loaded MSNs is urgently needed for minimizing the side effects and maximizing the therapeutic efficacy for lymphatic system tumors.

**Scheme 1:**
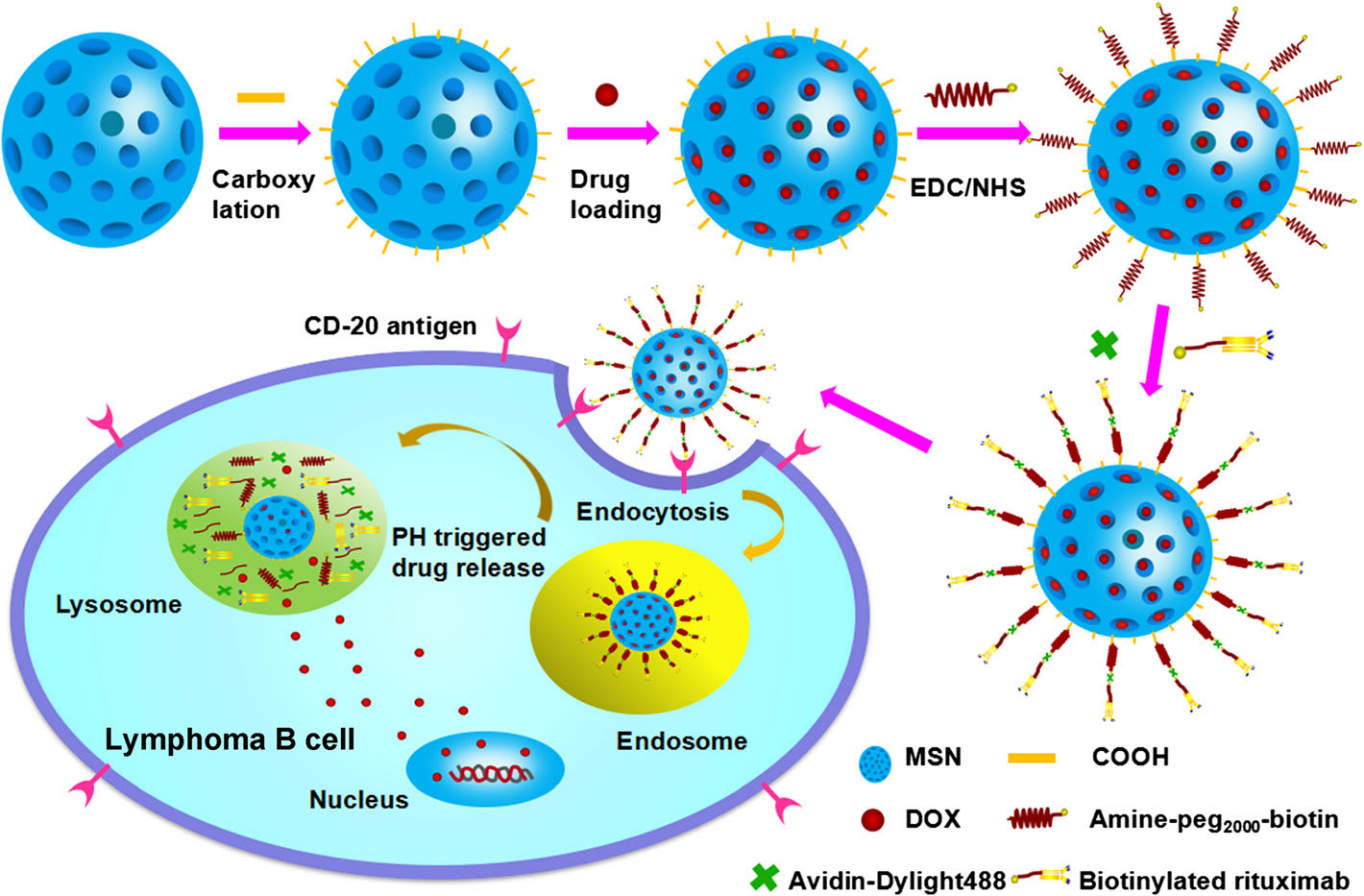
Self-assembly processes and structure of the RDMSNs and pH-responsive targeted drug delivery. Drug loading and rituximab conjugation processes of RDMSNs, and illustration of the uptake and internalization of the RDMSNs via a CD20 antigen-mediated interaction and pH-triggered DOX release in lysosome or endosome of the lymphoma B cells

In our previous study, rituximab was conjugated onto the surface of DOX - loaded microbubbles to improve targetability to CD20 receptor-overexpressed Raji cells, and rituximab conjugated and DOX-loaded microbubbles combined with ultrasound increased the release of DOX, resulting in enhanced antitumor efficacy [32]. In this study, the biocompatible poly (ethylene glycol) (PEG), which is a hydrophilic protection layer, can prevent functional MSNs from absorbing plasma proteins and being recognized by the reticuloendothelial system for clearance to allow for a prolonged blood circulation lifetime [33, 34]. Then, the functional DOX -loaded MSNs (DMSNs) were conjugated with the PEG. Moreover, rituximab was employed as a targeted ligand in the drug delivery system based on DMSNs. Therefore, we supposed that an intracellular pH-responsive and rituximab conjugated DOX-loaded MSNs (RDMSNs) was constructed for B cell lymphoma therapy. As mentioned above and envisioned in Scheme 1, in this paper we aim to evaluate whether (a) it is possible to design an intracellular pH-responsive and targeted drug delivery system based on MSNs, (b) targeted drug delivery system are able to selectively adhere to CD20 antigen positive lymphoma B cells and (c) whether molecules (i.e., DOX) can be selectively delivered to CD20 antigen positive lymphoma B cells upon binding with RDMSNs via the receptor-mediated endocytosis pathway for improving the therapeutic index.

## Methods

### Materials

Mesoporous silica nanoparticles were purchased from Anhui JingYe Nano Technology Co., Ltd. N-(trimethoxysilylpropyl) ethylenediamine triacetic acid was obtained from J&K Scientific Ltd. Doxorubicin hydrochloride was obtained from Shenzhen Wanle Pharmaceutical Co., Ltd. N-hydroxysuccinimide (NHS) was purchased from Aladdin Industrial Corporation. Amine-Peg2000-Biotin, the EZ-Link™ Sulfo-LC Biotinylation Kit and 1-ethyl-3-(3-dimethylaminopropyl) carbodiimide hydrochloride (EDC) were purchased from Thermo Fisher Scientific. Dylight 488-Avidin was obtained from Wuhan Boster Biological Engineering Co., Ltd. Rituximab was obtained from Hoffmann-La Roche, Ltd. The DAPI (4,6-diamidino- 2-phenylindole) staining solution, Cell Counting Kit-8 and the Annexin V-FITC/PI cell apoptosis detection kit were purchased from Beyotime Biotechnology Co., Ltd.

### Preparation of carboxyl-modified MSNs

The surfaces of the obtained MSNs were modified with carboxyl groups via hydrolyzation of N-(trimethoxysilylpropyl) ethylenediamine triacetic acid. Briefly, the MSNs (100 mg) were dispersed in anhydrous alcohol (20 mL), and 0.1 mL of N-(trimethoxysilylpropyl) ethylenediamine triacetic acid was added under continuous stirring with a magnetic stirring apparatus at room temperature for 24 h. Then, the resulting MSN-COOH were collected by repeated centrifugation and rinsed with a sterile PBS solution three times to remove the reaction impurities.

### Characterization

The morphological characterization of MSN-COOH was performed using a scanning electron microscope (SEM, Hitachi S-4800; Japan). The structures of MSN-COOH were investigated using transmission electron microscopy (TEM, Hitachi H-7600; Japan). The size distribution and zeta potential of the MSN-COOH resuspended in PBS (pH 7.4) were measured using a Malvern Zetasizer Nano ZS unit (Malvern Instruments, UK). Fourier transform infrared (FTIR) analyses of MSN and MSN-COOH were carried out on a Bruker Vertex 70 FTIR spectrometer. The pore size distributions and surface areas of different MSNs materials were characterized by Brunauer–Emmett–Teller (BET) and Barrett–Joyner–Halenda (BJH) analyses (ASAP2020M, USA).

### Drug loading

To load MSN-COOH with DOX, 5 mg of DOX hydrochloride was dissolved in 2 mL of PBS (pH 7.4), and MSN-COOH was added to the solution. The mixture was stirred on a magnetic stirring apparatus at room temperature for 24 h under dark condition. The DOX molecules can diffuse into the mesoporous channels with carboxyl groups. The dispersion was detached by centrifugation and washed with PBS to remove the unloaded DOX. The DMSNs were resuspended in PBS. The amount of supernatant DOX was estimated by absorbance measurement at 488 nm using a TECAN Infinite F50 Microplate Reader. Loading efficiency = (initial weight of DOX - supernatant weight of DOX)/weight of particles × 100%. Encapsulation efficiency = (initial weight of DOX - supernatant weight of DOX) / initial weight of DOX.

### Preparation of targeting DMSNs

The resulting DMSNs were dissolved in PBS, and an equimolar amount of EDC and NHS was added to the solution. The carboxyl groups on the surface of the DMSNs were activated for 1 h at room temperature in a shaker. The supernatant was removed after the mixture was centrifuged. The resulting precipitants were resuspended in PBS, and Amine-Peg2000-Biotin (5 mg) was added to the solution. Next, the mixture was allowed to react for 1 h at room temperature in a shaker to afford the biotinylated DMSNs. The biotinylated DMSNs were incubated with DyLight 488-labeled avidin for 10 min. The biotinylated DMSNs were collected by repeated centrifugation and rinsed three times to remove the redundant DyLight 488-labeled avidin. Then, rituximab was biotinylated through the EZ-Link™ Sulfo- LC-Biotinylation Kit according to the manufacturer’s instructions. Finally, biotinylated rituximab was added to the biotinylated DMSNs suspension and incubated for 10 min. The mixture was rinsed and purified to afford the RDMSNs. Rituximab-conjugated MSNs (RMSNs) were prepared using the same method but without DOX hydrochloride.

The conjugation of rituximab on the surface of the DMSNs was determined by measuring the absorbance of the free DyLight 488-avidin solution and DyLight 488-avidin conjugated DOX-loaded MSNs with a fluorescence spectrophotometer at a maximum excitation wavelength of 493 nm and a maximum emission wavelength of 518 nm. The binding efficiency of rituximab on the DyLight 488-avidin conjugated DMSNs was estimated as the ratio of the intensity of the DyLight 488-avidin conjugated DMSNs to the intensity of the free DyLight 488-labeled avidin samples.

### In vitro test of drug release

The DMSN and RDMSN samples were dissolved in 5 mL of PBS buffer (pH 5.5 or pH 7.4) or medium and shaken at 37 °C under dark conditions. The samples were centrifuged at certain intervals, and the amount of released DOX in the supernatant was determined using a TECAN Infinite F50 Microplate Reader.

### Cell culture

Raji and Daudi (CD20-positive) cell lines and Jurkat (CD20-negative) cell lines, which were purchased from the Type Culture Collection of the Chinese Academy of Sciences (Shanghai, People’s Republic of China), were cultured in RPMI 1640 medium with 10% FBS (v/v) and 1% PS (v/v) at 37 °C under a humid atmosphere containing 5% CO_2_ at 37 °C. The cells used in the experiments were in the logarithmic growth phase.

### Cell recognition and cellular uptake of RDMSNs and DMSNs

The Raji cells and Jurkat cells were seeded in a 12-well plate (50,000 per well) and incubated with RDMSNs and DMSNs with a DOX concentration of 0.5 μg/mL for 2 h at 37 °C. After the medium was removed and the cells were washed with PBS three times, flow cytometry (FCM) was employed to quantify the mean fluorescence intensity (MFI) of the RDMSNs and DMSNs internalized into the cells based on DOX autofluorescence. Additionally, the Raji cells and Jurkat cells were incubated with a nuclear staining agent (DAPI) in NEST glass culture dishes for 30 min followed by observation under a confocal laser scanning microscope (CLSM FV1000; Olympus, Japan) using a 100 × oil objective at excitation wavelengths of 410 and 488 nm, respectively. Competitive experiments were performed by pre-incubation of Raji cells with excessive rituximab to block the CD20 antigen for 30 min followed by washing. Jurkat cells were employed as a control.

Transmission electron microscopy was further employed to observe cell uptake and intracellular distribution of the nanoparticles. After the Raji cells were incubated with RDMSN and DMSN for 24 h, these cells were collected by repeated centrifugation and rinsed with PBS buffer. Then, the Raji cells were immobilized with 2.5% glutaraldehyde and observed using TEM.

### Cell viability assay of RDMSNs and DMSNs

The cell viability was measured according to the instructions provided with the Cell Counting Kit-8 (CCK-8). The Raji, Daudi and Jurkat cells were seeded in 24-well plates (1 × 10^5^ per well). The cells were incubated with various concentrations of MSNs or rituximab-conjugated MSNs (RMSNs) (i.e., 10, 20, 40, 80, or 100 μg/mL), Free DOX, RDMSNs and DMSNs containing various DOX concentrations (i.e., 0.1, 0.5, 1.0, 2.0, or 4.0 μg/mL). After incubation for 24 h, the cells were washed with PBS buffer three times and placed in fresh culture medium. Then, 10 μL of the CCK-8 solution were added to each well followed by incubation for an additional 4 h. The absorbance of the solution was measured at 450 nm using a TECAN Infinite F50 Microplate Reader.

### Cell apoptosis assay of RDMSNs and DMSNs

The nuclear morphological changes due to apoptosis were detected in Raji cells using CLSM. The Raji cells were seeded in a 6-well plate (4 × 10^5^ per well) and incubated with PBS buffer, MSNs (50 μg/mL), Free DOX, DMSNs and RDMSNs containing a DOX concentration of 2.0 μg/mL for 24 h. Next, the cells were rinsed with cold PBS buffer three times and fixed with 4% paraformaldehyde at room temperature for 30 min. Then, the samples were washed with PBS and stained with DAPI (20 μg/mL) in NEST glass culture dishes for 30 min. Finally, the stained Raji cells were observed under CLSM.

The cell apoptosis assay was analyzed by FCM with a commercial Annexin V-FITC detection kit. The Raji cells were treated using the same method. In addition, the Raji cells were incubated with different concentrations of RDMSNs (i.e., 0, 10, 20, 30, and 50 μg/mL). After incubation for 24 h, the Raji cells were rinsed twice with cold PBS buffer, resuspended in 195 μl of binding buffer solution and stained with 5 μl of FITC-labeled Annexin V and 10 μl of PI for 15–20 min at room temperature in the dark. Raji cells incubated in PBS buffer were considered the control group.

### RMSNs biological safety study in vivo

Female Babl/c mice were treated with RMSNs by tail vein injection (100 μL, 100 mg/kg) one dose every 3 days for 3 weeks. The nude mice treated with saline were used as controls (3 mice per group). The body weight was monitored every week. The main organs including the heart, liver, spleen, lungs and kidneys were acquired after the final treatment followed by Hematoxylin and eosin (H&E) staining to detect the toxicity in vivo.

### DOX distribution in tumors

Lymphoma model were established by subcutaneous injection with 6 × 10^7^ Raji cells into the right axillary space of mice. When lymphoma volume reached approximately 100 mm^3^, the intravenous injection of Free DOX, DMSNs and RDMSNs was then carried out at the DOX dose of 2 mg / kg at 1, 6, and 24 h, respectively (9 mice per group). After the sacrifice of 3 mice per group for each time points, the tumors were excised and homogenized with RIPA buffer. Subsequently, the supernatant was collected by centrifugation and quantified for DOX content by fluorimetry and normalized with the tumor weight.

### In vivo antitumor efficacy

Raji lymphoma bearing mice were established as described above. When lymphoma volume reached approximately 100 mm^3^, Raji lymphoma bearing mice were randomly divided into 4 groups (5 mice per group), respectively : saline group, Free DOX group, DMSNs group and RDMSNs group. Each Raji lymphoma bearing mouse was treated with DOX related formulations (the dose was 2 mg / kg) once every 4 days for a total four times. The lymphoma sizes of all mice were monitored by a digital caliper and calculated by the equation: V_tumor_ = LW^2^/2 (L: tumor length, W: tumor width). The lymphoma volume and body weight were determined every other day. After 16 days of treatment, all the mice were sacrificed and the lymphoma were extracted and fixed with 4% paraformaldehyde. Additionally, To detect cell apoptosis in lymphoma tissue, lymphoma tissue was sliced into thin sections and stained with a TUNEL apoptosis detection kit. Next, the sample was stained with DAPI solution to visualize cell nuclei under a fluorescence microscope.

### Statistical analysis

All experiments were performed in triplicate. Results were presented as mean ± standard deviation and analyzed using SPSS 16.0 software (SPSS, Chicago, IL, USA). Statistical analysis was carried out using a student’s *t*-test. *P* < 0.05 was determined to be statistically significant.

## Results

### Preparation and characterization of nanoparticles

**Fig. 1:**
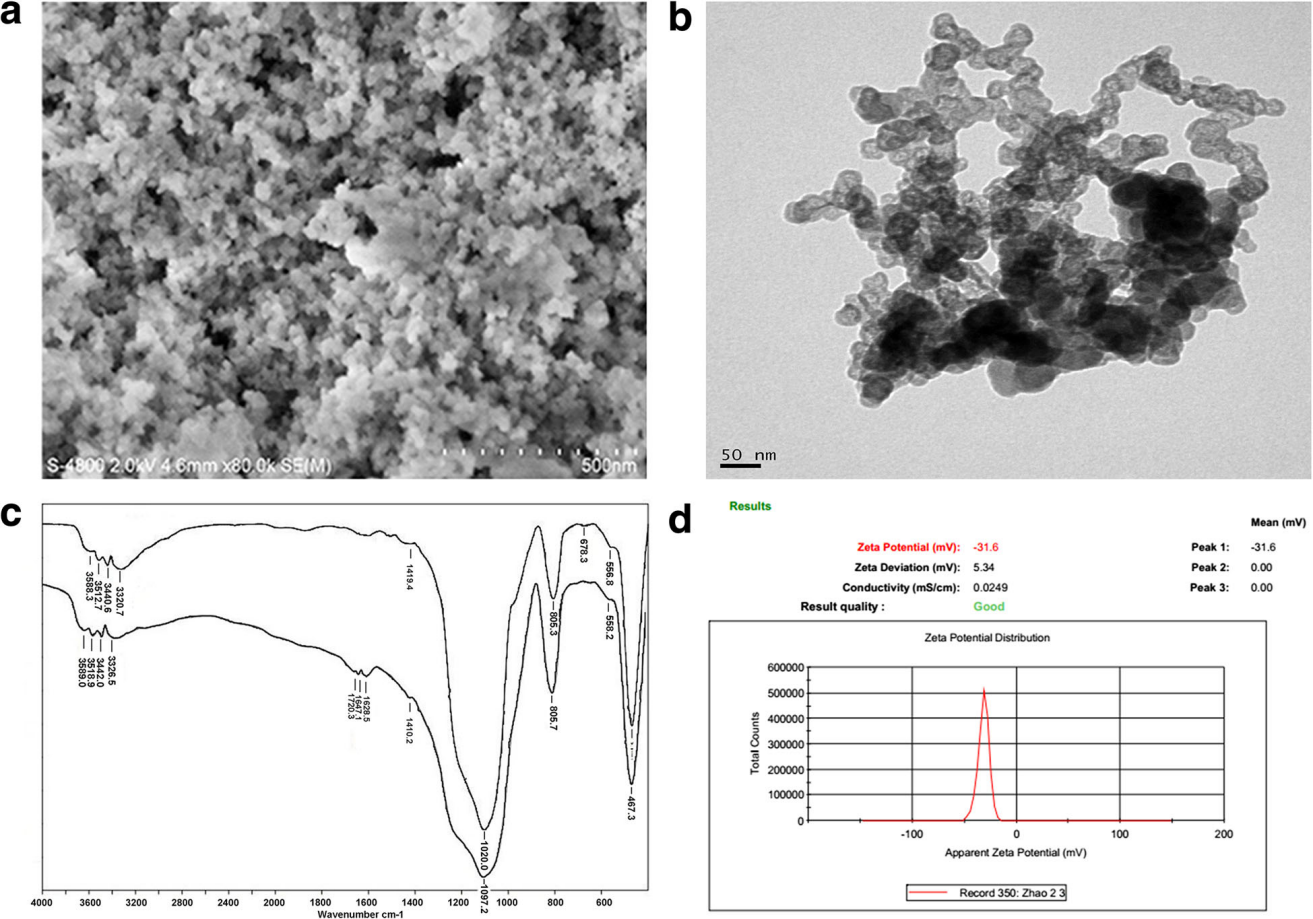
Representative characterizations of MSN and MSN-COOH. SEM images of MSN-COOH, *scale bar*: 500 nm (**a**), TEM images of MSN-COOH, *scale bar*: 50 nm (**b**), the FT-IR spectra of MSN and MSN-COOH (**c**), and the zeta potential of MSN-COOH (**d**)

Scheme 1 shows the synthesis of DMSNs and RDMSNs. RDMSNs were internalized by Raji cells via CD20 antigen-mediated endocytosis and then DOX was released into tumor cells by a pH trigger, which enhanced intracellular drug accumulation. The size distribution of the MSN-COOH was uniform and regular (Fig. 1a), and the MSN-COOH possessed well-defined global morphology (Fig. 1b). Furthermore, based on the FTIR spectra, MSN-COOH exhibited an absorption peak at 1647–1720 cm^−1^, which characteristic of the C = O stretching vibrations of carboxyl groups (Fig. 1c). The mean diameter of MSN-COOH was in the range of 40.7 ± 19.1 nm. The zeta potential of MSN-COOH was −32.6 ± 5.7 mV (Fig. 1d). These results indicated that MSNs can serve as drug delivery carriers.

### Characterization of nanoparticles drug loading and release

To investigate the drug loading behavior of MSNs, an anticancer drug (i.e., DOX) was chosen as a model drug. The properties of the MSNs as a drug delivery system were estimated by the DOX loading and encapsulation efficiencies. The encapsulation efficiency was approximately 45.2 ± 3.1%, and the loading efficiency was approximately 23.5 ± 1.3%. The data are similar to that of the previous study [35]. These results demonstrated that the MSNs possessed a large storage space for the effective loading of DOX.

**Fig. 2:**
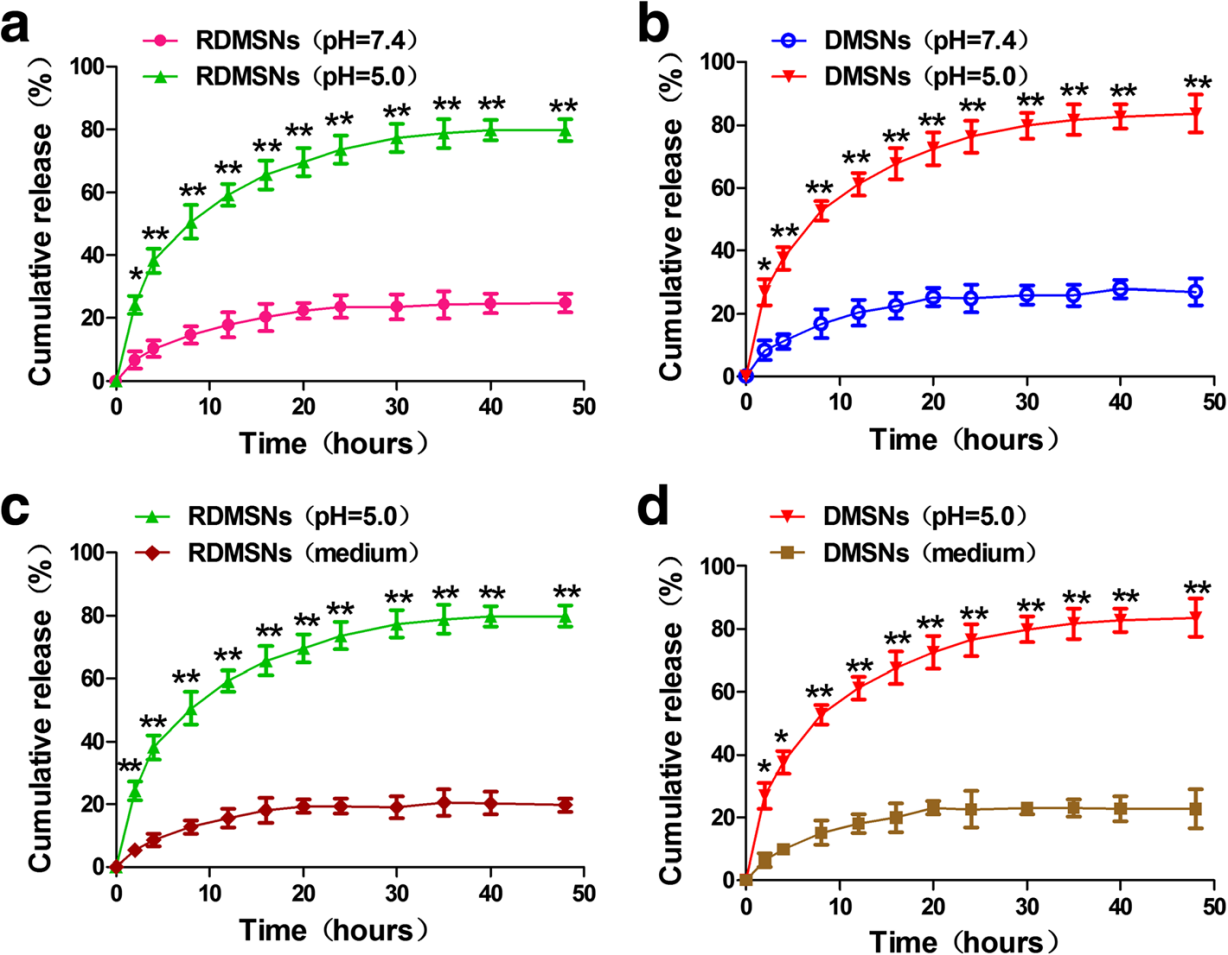
pH-responsive release profiles of DOX from the DMSNs and RDMSNs in medium and PBS solution (*pH 5.0* and *pH 7.4*) over 48 h, respectively. These results indicated that the DOX released from nanoparticles was more at a pH of 5.0 than that at a pH of 7.4 or medium. RDMSNs (*PBS pH7.4*) vs RDMSNs (*PBS pH5.0*) (**a**), DMSNs (*PBS pH7.4*) vs DMSNs (*PBS pH5.0*) (**b**), RDMSNs (*PBS pH 5.0*) vs RDMSNs (*medium*) (**c**), and DMSNs (*PBS pH 5.0*) vs DMSNs (*medium*) (**d**). Data are presented as mean ± SD from three independent experiments. *, *P* < 0.05; **, *p* < 0.01

Size distribution and zeta potential of RDMSNs were 56.3 ± 11.2 nm and −31.5 ± 5.2 mV, respectively, and there were no significant change compared to the MSNs, indicating that the DOX payload in MSN-COOH as well as the conjugation of rituximab on the surface of the MSNs slightly altered the particle size and surface charge of the MSNs (Table 1). To investigate the pH-responsive drug release property, a release assay of DMSNs and RDMSNs was carried out under pH 7.4, pH 5.0 conditions in vitro. Only a DOX loading of 24.8 ± 6.4 and 23.7 ± 5.1% was released from the DMSNs and RDMSNs, respectively, at a pH of 7.4. However, a DOX loading of approximately 76.4 ± 7.2 and 73.6 ± 6.2% was released from the DMSNs and RDMSNs at a pH of 5.0, respectively. The DOX released from nanoparticles was significantly different at a pH of 5.0 compared with that at a pH of 7.4 (Fig. 2). Additionally, the drug release properties of DMSNs and RDMSNs in medium were also estimated and the results were similar to those at a pH of 7.4 condition. Therefore, this drug delivery system possessed pH-responsive drug release property.

**Table 1:** Characterization of the MSNs

Groups	Size distribution (nm)	Zeta potential (mV)
MSN-COOH	40.7 ± 19.1	−32.6 ± 5.7
DMSNs	45.3 ± 17.6	−27.1 ± 3.8
RDMSNs	56.3 ± 11.2	−31.5 ± 5.2

All data were recorded by dynamic light scattering. Values are means ± SD (*n* = 3)

**Fig. 3:**
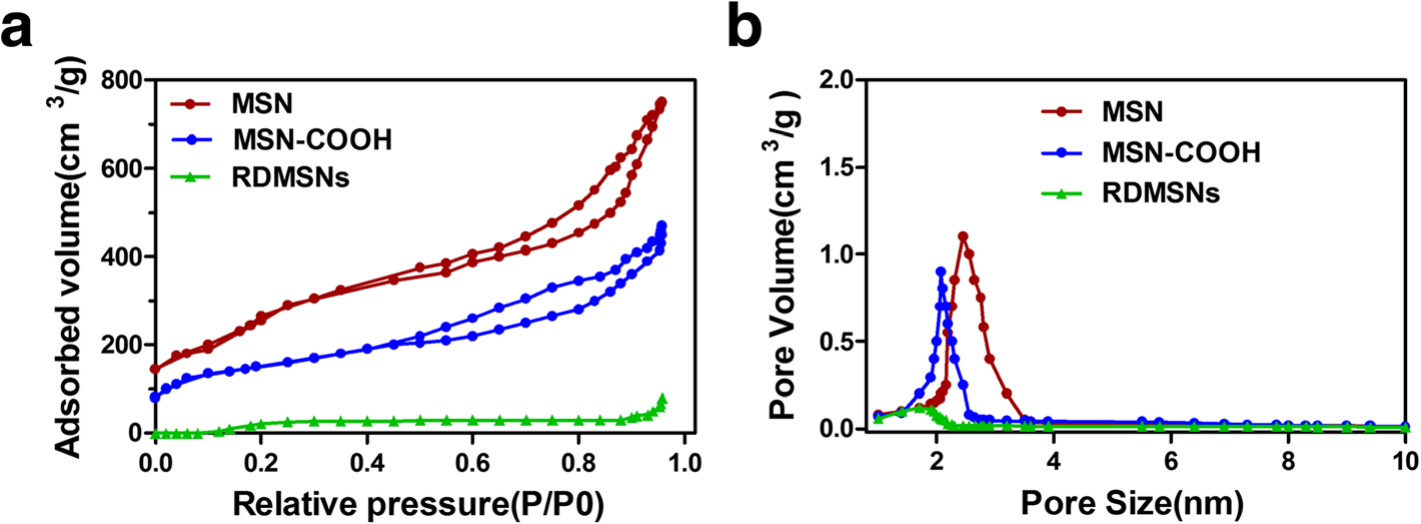
The characterizations of nanoparticles. BET nitrogen adsorption /desorption isotherms (**a**), and BJH pore size distribution of MSN, MSN-COOH and RDMSNs. After carboxyl grafting and cargo loading, the pore volume and pore diameter of nanoparticles decreased, indicating that the nanopores were blocked by the incorporated DOX and rituximab molecules (**b**)

The mesoporous structure of the MSN was further confirmed by the nitrogen adsorption/desorption isotherm. The MSNs have well-defined mesoporous nanopores with a surface area of 820 m^2^/g (Fig. 3a), a pore volume of 0.62 cm^3^/g and an average diameter of 2.5 nm (Fig. 3b). After carboxyl grafting and cargo loading, the pore volume and pore diameter of nanoparticles decreased, indicating that the nanopores were blocked by the incorporated DOX and rituximab molecules.

**Fig. 4:**
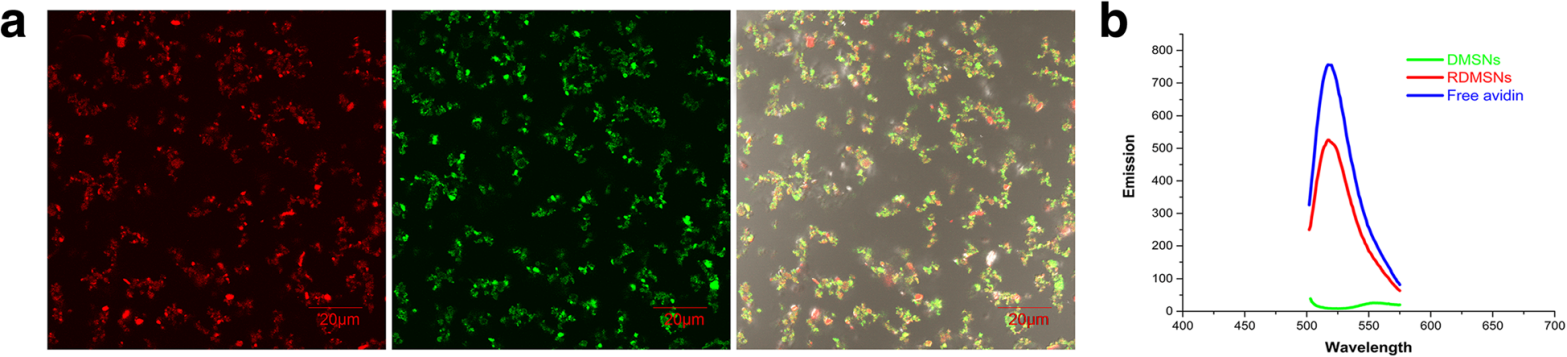
The fluorescence characterizations of nanoparticles. Imaging of MSNs was performed by CLSM (100 × objective) the loaded DOX imaging of the RDMSNs (*red fluorescence*), DyLight 488-avidin imaging of the RDMSNs (*green fluorescence*), merge imaging of RDMSNs **a**. The fluorescence absorbance of the RDMSNs, DMSNs and free DyLight 488-avidin were measured to analyze the rituximab conjugation efficiency. The fluorescence intensity of the RDMSN suspensions was 66.25% for the DyLight 488-labeled avidin samples, demonstrating the desired conjugation efficiency of biotinylated rituximab to the avidin-conjugated RDMSNs **b**

Subsequently, to estimate the rituximab conjugation efficiency, the quantities of rituximab on the surface of the RDMSNs were determined by examining the fluorescence intensity of the RDMSN suspensions. Fluorescence imaging of the RDMSNs revealed a dense layer of green under CLSM, which confirmed the conjugation of DyLight 488-labeled avidin onto the surface of the RDMSNs (Fig. 4a). The fluorescence intensity of the RDMSN suspensions was 66.25% for the DyLight 488-labeled avidin samples, demonstrating the desired conjugation efficiency of DyLight 488-labeled avidin to the biotinylated DMSNs (Fig. 4b). Due to the high affinity of avidin to biotin, we indirectly deduced the same high linkage of biotinylated rituximab to the avidin-conjugated RDMSNs. Therefore, the results showed that the multifunctional MSNs could serve as a targeted drug delivery system for B cell lymphoma therapy.

### Cell recognition and internalization of DMSNs and RDMSNs

**Fig. 5:**
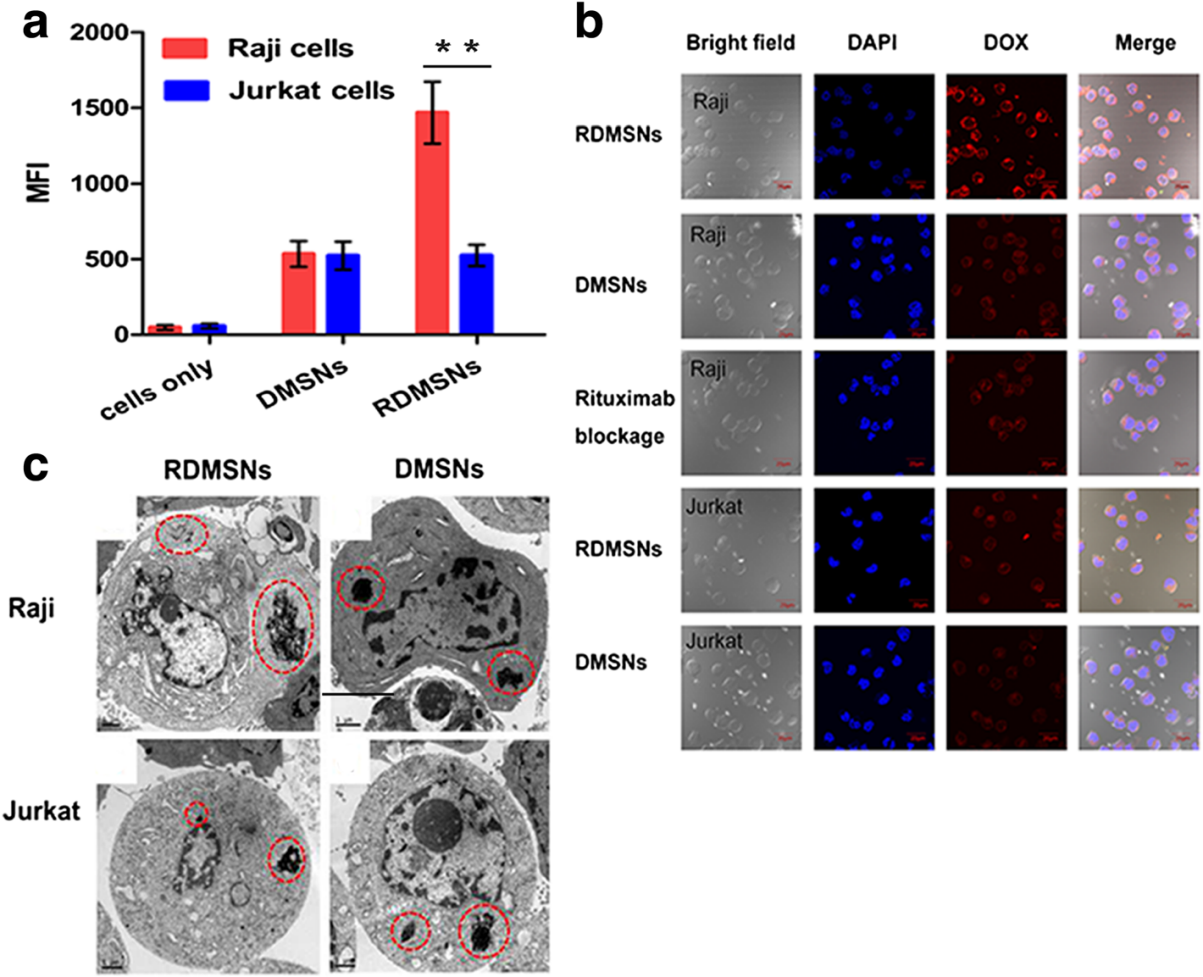
The recognition and internalization of the DMSNs and RDMSNs in Raji and Jurkat cells, respectively. FCM analysis denotes the mean fluorescence intensity of DOX inside Raji treated with RDMSNs was much higher compared to that of Jurkat cells. Data are presented as mean ± SD from three independent experiments. **, *p* < 0.01 (**a**). The Raji cells that were incubated with RDMSNs exhibited strong DOX fluorescence in the cytoplasm. In contrast, the DOX fluorescence that was observed in the cytoplasm of Raji cells incubated with DMSNs was very weak. The cell nucleus were stained with DAPI (*blue*) (**b**). TEM images showing the distribution of the RDMSNs within Raji was more compared to that of Jurkat cells. Moreover, more RDMSNs were internalized by Raji cells compared to DMSNs (**c**)

To evaluate the tumor cell targetability of RDMSNs in vitro, the amount of cellular internalization of DMSNs and RDMSNs was analyzed using FCM. The MFI values of the Raji cells treated with RDMSNs and DMSNs at 37 °C for 2 h were 1445 and 543, respectively (Fig. 5a). Moreover, the cellular recognition and internalization behavior of DMSNs and RDMSNs were investigated using CLSM in Raji and Jurkat cells, respectively. The Raji cells that were incubated with RDMSNs exhibited strong DOX fluorescence in the cytoplasm. In contrast, the DOX fluorescence that was observed in the cytoplasm of Raji cells incubated with DMSNs was very weak. In the rituximab blockage experiment, the resulting outcome was indicated by weak DOX fluorescence in the cytoplasm of Raji cells. In addition, only a weak DOX fluorescence was observed in Jurkat cells incubated with RDMSNs and DMSNs (Fig. 5b). Furthermore, we used TEM to investigate the distribution of DMSNs and RDMSNs after endocytosis in vitro. RDMSNs and DMSNs aggregated only in the cytoplasm but did not penetrate into the nucleus. Importantly, more RDMSNs were internalized by Raji cells compared to DMSNs. In the negative control, the number of endocytosed RDMSNs by Jurkat cells was similar to that of DMSNs (Fig. 5c). Thus, these outcomes hinted that this targeted drug delivery system can significantly more be recognized and internalized by lymphoma B cells compared to nontargeted drug delivery system.

### Cell viability study of DMSNs and RDMSNs

**Fig. 6:**
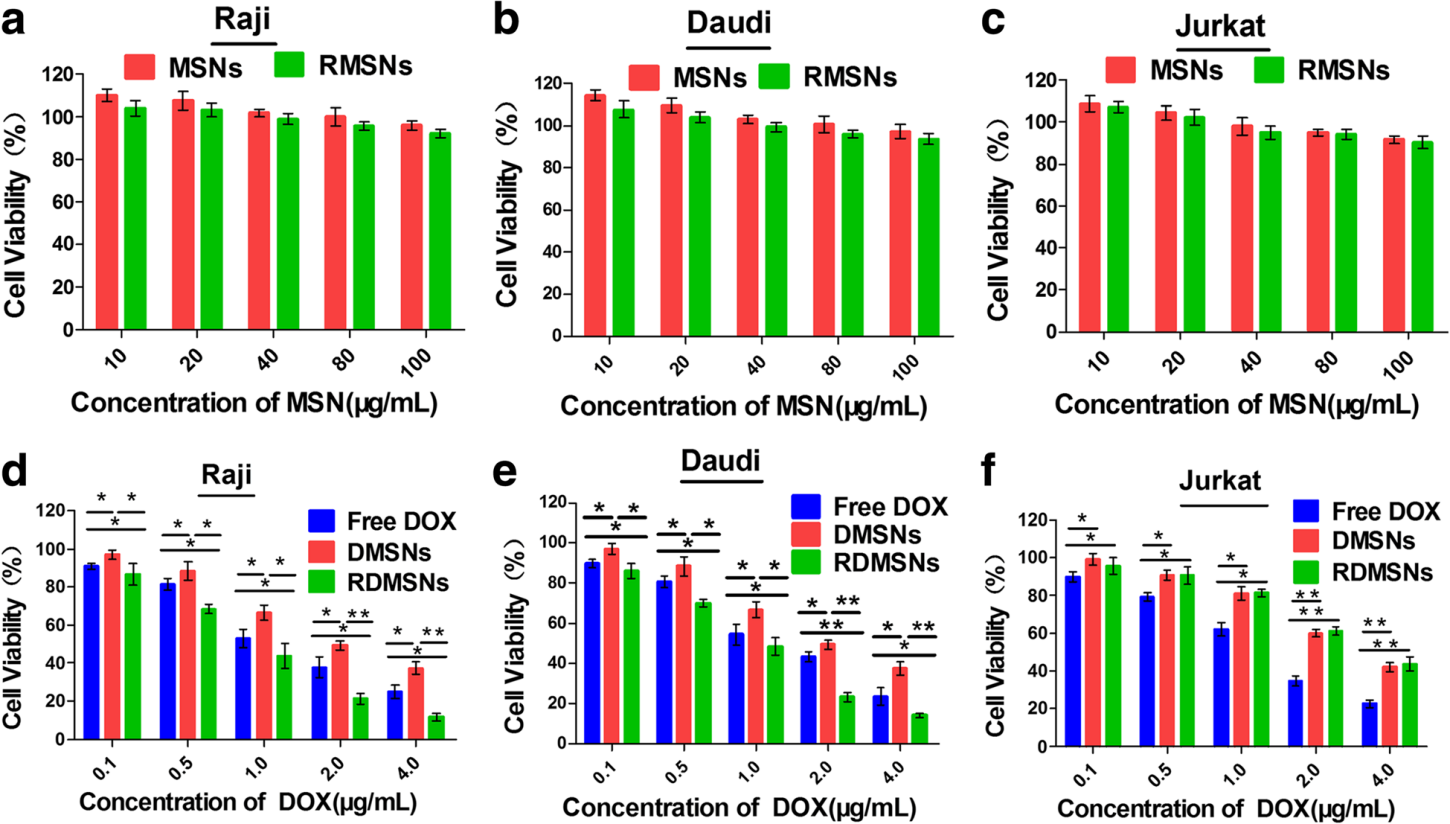
The cytotoxicity of different formulations in Raji, Daudi, and Jurkat cells. Cytotoxicity of MSNs and RMSNs on Raji cells (**a**), Daudi cells (**b**) and Jurkat cells (**c**). The cell viability of Raji cells treated with 100 μg/mL of MSNs or RMSNs was as high as 90%, implying that MSNs and RMSNs have good biocompatibility. Cytotoxicity of Free DOX, DMSNs and RDMSNs on Raji cells (**d**), Daudi cells (**e**) and Jurkat cells (**f**). Raji and Daudi cells treated with RDMSNs exhibited higher cytotoxicity compared to that of Free DOX, while Jurkat cells treated with RDMSNs exhibited lower cytotoxicity compared to that of Free DOX at the same DOX concentration. Data are presented as mean ± SD from three independent experiments.*, *P* < 0.05; **, *p* < 0.01

Next, To estimate the cytotoxicity of MSNs, RMSNs, DMSNs and RDMSNs, a CCK-8 assay was performed to quantify the cell viability of Raji, Daudi and Jurkat cells. Figure 6a showed the cell viability of Raji cells after being incubated with a series of concentrations of MSNs or RMSNs for 24 h. The cell viability of Raji cells treated with 100 μg/mL of MSNs was as high as 90%. Moreover, similar results were obtained for the Daudi and Jurkat cells (Fig. 6b and c). These experimental data demonstrate that MSNs and RMSNs exhibit good biocompatibility. The cell viability of Raji cells that were treated with RDMSNs, DMSNs and Free DOX was also determined in this study. When incubated with Raji cells, both the Free DOX and DMSNs exhibited lower antitumor activity compared to that of RDMSNs at the same DOX concentration. Although both Free DOX and DMSNs exhibited dose-dependent toxicity to the Raji cells, Free DOX had much stronger cytotoxicity compared to that of DMSNs (Fig. 6d). Similar results were obtained for the Daudi cells (Fig. 6e). Additionally, a Jurkat cell control group was also investigated. Jurkat cells incubated with RDMSNs and DMSNs exhibited lower cytotoxicity compared to that of Free DOX (Fig. 6f). The viability of Jurkat cells of the RDMSN and DMSN groups exhibited no significant difference. Moreover, all cells incubated with RDMSNs, DMSNs and Free DOX exhibited a DOX dose-dependent cytotoxicity at a series of DOX concentrations ranging from 0.1 to 4.0 μg/mL. These results confirm that the prepared RDMSNs exhibit a significant selective cytotoxicity effect on CD20 antigen overexpressed tumor cells and can reduce the side effects on normal or healthy cells.

### Cell apoptosis study of DMSNs and RDMSNs

**Fig. 7:**
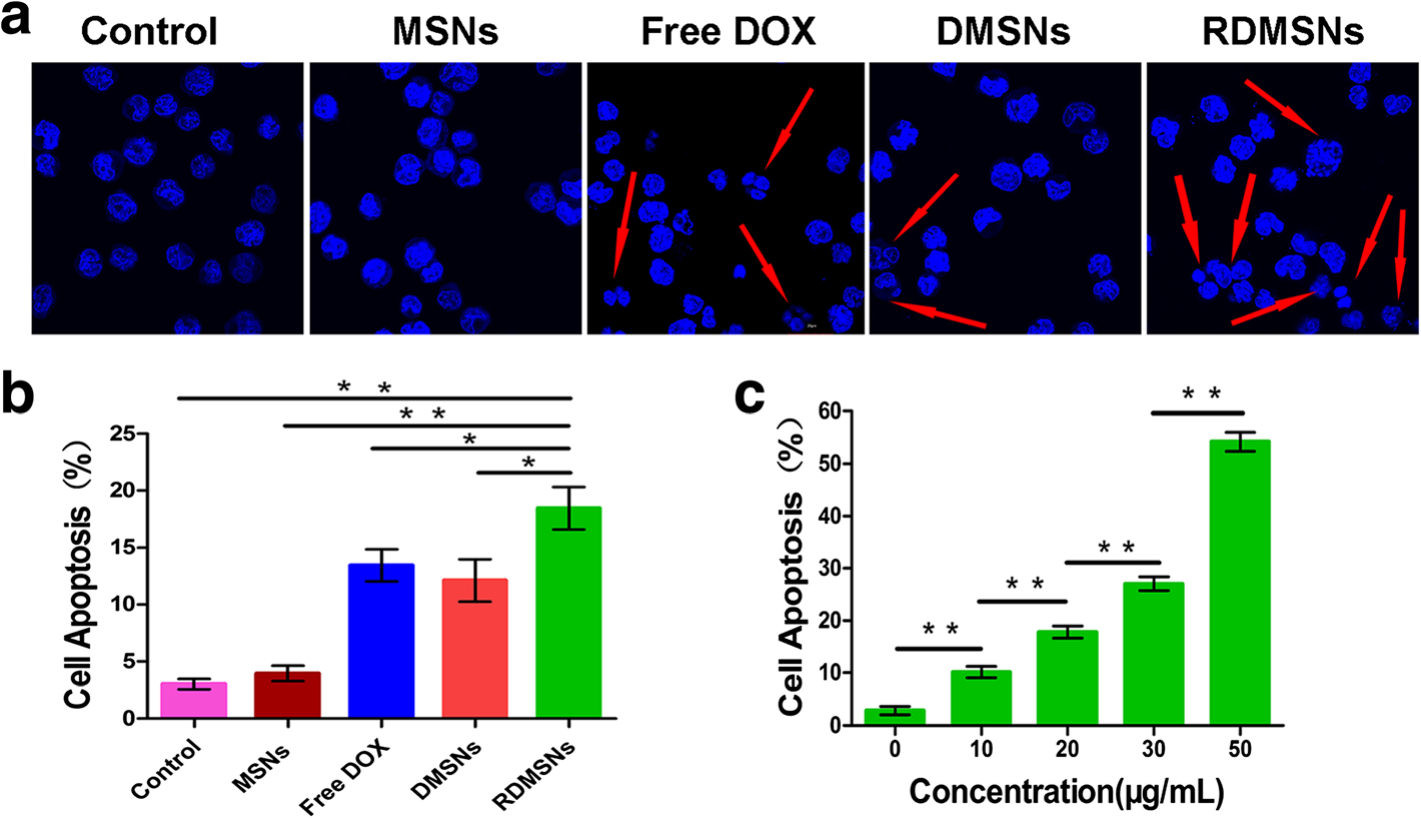
Raji cells apoptosis characterizations in vitro. Nuclei morphologic characterizations of Raji cells after different treatment for 24 h at 37 °C under CLSM (1000×, DAPI). The Raji cells that were treated with RDMSNs exhibited the most significantly irregular cell nuclei morphology with shrinkage features compared to those of other groups. Note: *Red arrows* direct characteristic variations of nucleus (**a**). Apoptotic rate of Raji cells treated for 24 h at 37 °C by FCM. The apoptosis efficiency of Raji cells that were incubated with RDMSNs was significantly higher than those of other groups (**b**). Quantitative apoptosis analysis of Raji cells treated with various concentrations of RDMSNs for 24 h at 37 °C. *P < 0.05. The concentration-dependent apoptosis efficiency was detected (**c**). Data are presented as mean ± SD from three independent experiments.*, *P* < 0.05; **, *p* < 0.01

To investigate Raji cell apoptosis after treatment with MSNs, DMSNs, RDMSNs and Free DOX for 24 h, the morphologies of the nucleus associated with apoptosis were observed using CLSM. The cell apoptosis analysis was also investigated using FCM. The Raji cells that were treated with MSNs demonstrated a similar elliptic nuclear morphology with intact profiles. The Raji cells that were treated with RDMSNs exhibited the most significantly irregular cell nuclei morphology with shrinkage features compared to those of Raji cells treated with DMSNs and Free DOX (Fig. 7a). The late apoptosis or necrosis of Raji cells treated with PBS, MSNs, Free DOX, DMSNs and RDMSNs for 24 h was 3.03 ± 0.36, 3.94 ± 0.54,18.22 ± 1.15,10.09 ± 1.1 and 23.29 ± 1.37%, respectively. From these experimental data, the late apoptosis or necrosis of Raji cells that were incubated with RDMSNs was significantly higher than those of Raji cells incubated with DMSNs and Free DOX (Fig. 7b). Moreover, we also detected the concentration-dependent apoptosis efficiency caused by the RDMSNs. After treatment for 24 h, Raji cell apoptosis increased when the concentration of the RDMSNs increased (Fig. 7c). These findings demonstrated that this targeted drug delivery system could induce lymphoma B cells apoptosis.

### RMSNs biological safety study in vivo

**Fig. 8:**
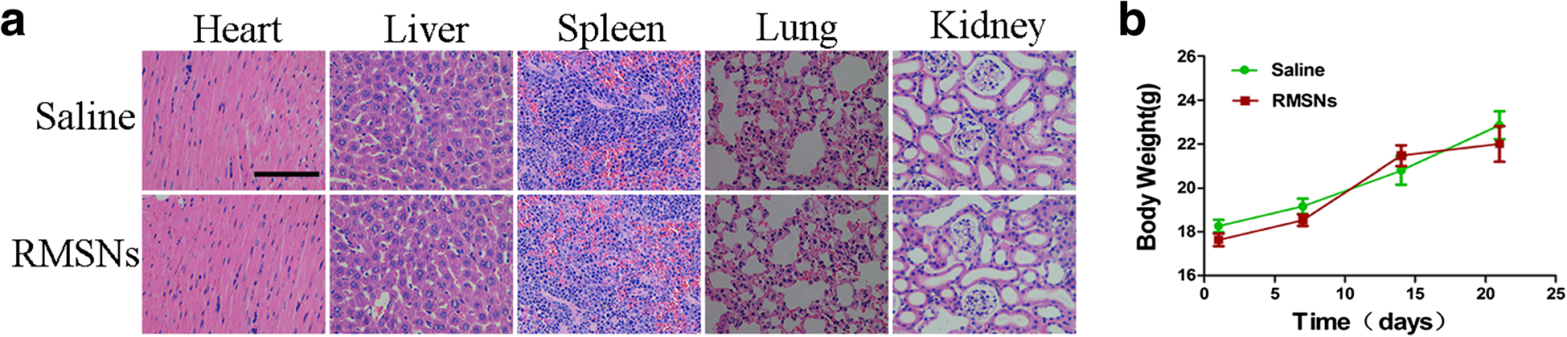
In vivo biological safety study. H&E staining of major organs obtained from nude mice treated with saline and RMSNs for 3 weeks, respectively. There was no significant pathological lesions or damages in the major organs from nude mice that were treated with RMSNs. *Scale bar*: 100 μm (**a**). Body weight of nude mice after treatment with saline and RMSNs showed a gradually increased tendency, implying that RMSNs had a good biocompatibility (*n* = 3) (**b**)

The potential in vivo toxicity of MSNs for drug delivery system is always of great concern. For safety purpose, we evaluated the in vivo toxicity of the drug carrier in nude mice treated with RMSNs by tail vein injection. We completed the histological analyses, which indicated no significant pathological lesions or damages in the major organs from nude mice that were treated with RMSNs for 3 weeks (Fig. 8a). Additionally, the increase in body weight of the RMSNs and saline groups showed a similar tendency over the 3 weeks (Fig. 8b). These results demonstrated that RMSNs had a good biocompatibility.

### Enhanced DOX accumulation in tumors and antitumor efficacy in vivo

**Fig. 9:**
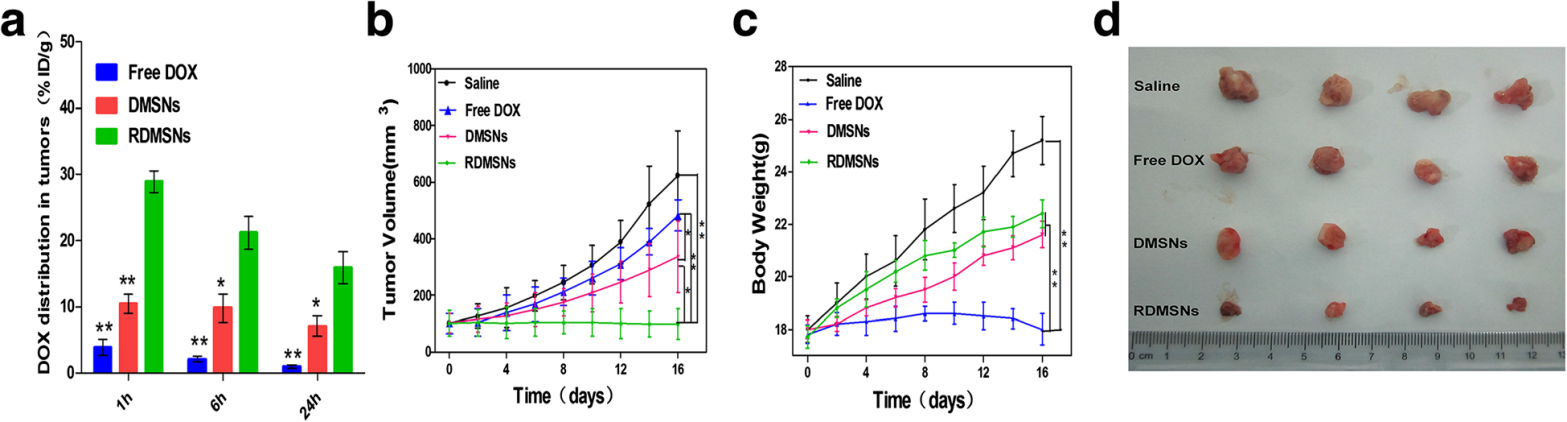
In vivo tumorous distribution and therapeutic effect of RDMSNs. The content of DOX in tumors treated with RDMSNs was much higher compared with those of other groups at different time points (*n* = 3). *P < 0.05,** *P* < 0.01 vs RDMSNs (**a**). Tumors treated with RDMSNs grew slowly compared to those of other groups. From the 10th day, the mean tumor volume of mice treated with RDMSNs was significantly different compared with those of other groups (**b**). The mice weight increased gradually with different degrees after injections of saline, DMSNs, and RDMSNs, respectively, while the mice weight in Free DOX groups showed a gentle reduction from the 8th day, indicating that Free DOX had severe systemic toxicity on nude mice. From the 10th day, the mean body weight of mice treated with Free DOX was significantly different compared with those of other groups (**c**). After 16 days culture, the volumes of tumors treated with saline were significantly larger compared to those of other groups. In contrast, The mice treated with RDMSNs exhibited significant inhibition of tumor growth in volume (**d**) (*n* = 5,*P < 0.05,** *P* < 0.01)

To investigate RDMSNs targeting capacity in vivo, the content of DOX in tumor was examined at 1, 6, and 24 h post injection in Raji cells grafted mice. As depicted in Fig. 9a, the DOX distribution of tumors in all groups was slowly reduced with time extending from 1 to 24 h post injection. In spite of this declining tendency, it was observed that RDMSNs and DMSNs displayed much higher DOX accumulation at each time point than Free DOX. Importantly, RDMSNs exhibited obviously improved content of DOX in tumor compared to that of DMSNs, implying that the potent in vivo tumor targeting of RDMSNs might be caused by the special binding of antibodies and antigens of cell membrane.

**Fig. 10:**
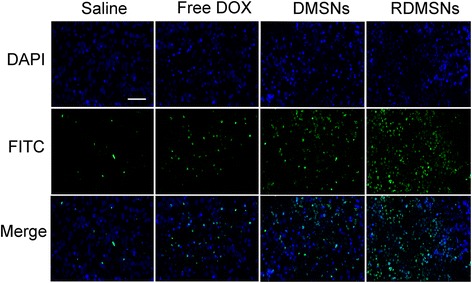
TUNEL assays showed that tumors treated with RDMSNs exhibited significantly more apoptosis DNA compared to those of other groups. Histological apoptosis observation in tumor tissues by TUNEL staining after the tumor-bearing mice were treated with Saline, Free DOX, DMSNs, and RDMSNs for 16 days, respectively. *Green*: apoptosis DNA; *Blue*: cell nuclei. *Scale bar*: 50 μm

To detect the therapeutic efficacy of RDMSNs, we intravenously injected mice bearing lymphoma with RDMSNs once every 4 days for 4 times. RDMSNs indicated the strongest antitumor efficacy among all groups. Furthermore, the tumor growth inhibition of DMSNs group was higher than those of Free DOX group. Interestingly, unlike the results of the cytotoxic effects evaluation in vitro, Free DOX indicated a slight inhibition effect on tumor growth in vivo. More importantly, RDMSNs group showed stronger antitumor efficacy compared to that of DMSNs group (Fig. 9b). After the last injection, the average tumor volume of RDMSNs group was much smaller than that of other groups, which was further demonstrated through visual observation (Fig. 9d). Alteration of body weight is an indication of systemic toxicity. To explore the potential systemic toxicity of RDMSNs in vivo, the body weight of nude mice was also periodically monitored. The mice weights in Free DOX groups showed a continuous and slow reduction from the 8th day, indicating that DOX had severe systemic toxicity on nude mice. However, the mice weight increased gradually with different degrees after injections of saline, DMSNs, and RDMSNs, respectively, which could be attributed to the low toxicity of DOX at injection dosage (Fig. 9c). Additionally, to detect the potential mechanism of lymphoma growth inhibition by this drug delivery system, TUNEL apoptosis assay was carried out to evaluate the antitumor efficacy. Sparse cell apoptosis (green fluorescence) was observed in groups treated with saline. The lymphoma tissues of mice treated with DMSNs and Free DOX displayed moderate cell apoptosis (green fluorescence), while those treated with RDMSNs exhibited the most significant apoptosis among all groups (Fig. 10). These results firmly verified that RDMSNs could inhibit tumor growth via inducing cells apoptosis in lymphoma. Therefore, RDMSNs could be used to reduce the nonspecific toxicity of DOX and realize the clinical application of some potent antitumor agents.

## Discussion

In the war against lymphoma, chemotherapy is presently the most common treatment. However, its efficiency is hampered by severe toxic side effects. These adverse effects often lead to a drug dose reduction or discontinuation of treatments in clinical applications. Nanoparticles as excellent drug delivery carriers have been reported to serve as an effective approach to enhance the drug efficacy and reduce adverse effects [36, 37]. The drug-loaded MSNs have been reported to serve as a promising drug carrier for all types of solid tumors therapy. However, the ligands conjugated to targeted drug-loaded MSNs had the unwanted immunogenicity. In this study, we successfully designed and constructed an intracellular pH-responsive and targeted drug delivery system based on DOX-loaded MSNs to enhance the therapeutic index and reduce the side effects of B cell lymphoma therapy.

The prepared RDMSNs have several advantages. First, PEG as a polymer protective layer could suppress plasma protein adsorption. Furthermore, rituximab as tumor-targeting components could carry out CD20 antigen-mediated cell internationalization. Additionally, DOX-loaded RDMSNs could be released by a low pH signal at the targeted site. Thus, after the multifunctional RDMSNs reach their targeted sites, the rituximab on the outer layer can specifically adhere to the CD20 antigen on the membranes of lymphoma B cells, leading to receptor-mediated cell pinocytosis or endocytosis and endosome or lysosome formation inside the tumor cells. Then, the loaded drug can be rapidly released from the multifunctional RDMSNs due to a reduction of the electrostatic interaction triggered by the lower pH of the acidic environment of the endosome or lysosome. Therefore, these multifunctional RDMSNs have the potential for use as an intracellular pH-responsive and targeted drug delivery system for B cell lymphoma therapy.

In this study, the zeta potential results demonstrated that the MSNs were negatively charged at a pH of 7.4 due to the large amount of negatively charged silanol group on the MSNs, which is consistent with that reported in a previous study [38]. Moreover, several studies confirmed that the isoelectric point (pI) of DOX is 8.2. It is important to note that the pI values of DOX are higher than pH 7.4, revealing that the DOX molecules are positively charged in the physiological microenvironment. Therefore, the DOX molecule can fill the mesoporous channels of the MSNs under pH 7.4 conditions via electrostatic interactions between the negative charges of the MSNs and the positive charges of the DOX molecules. Thus, the DMSNs further exhibit high loading and encapsulation efficiencies. In the drug release assay, no significant difference in the amount of released DOX was observed between the DMSNs and RDMSNs at the same pH value. Furthermore, the conjugation of rituximab did not influence the release of DOX at the different pH values. Interestingly, the release efficiency of DOX at a pH of 5.5 was higher than that at pH of 7.4 or in medium, indicating that a lower pH value was more beneficial to DOX release from the DMSNs or RDMSNs. These results were supported by those from previous studies [39]. The pH-responsive DOX release was due to the weakly electrostatic attraction at acidic pH and the strongly electrostatic attraction at physiological pH between MSNs and DOX molecules. Due to this property, the pH-responsive and targeted drug release system could effectively decrease DOX release during blood circulation (pH 7.4) and improve DOX release in the endosomes or lysosomes (pH 4.5–5.5) of tumor cells. Therefore, reduced DOX side effects and enhanced intracellular DOX accumulation could be achieved via this pH-responsive and targeted drug delivery system.

Previous studies reported similar results whereby a targeted modification of ligand enhanced the internalization and cytotoxicity effect of nanoparticles via the receptor-mediated endocytosis pathway [40–42]. In our study, the results demonstrated that more RDMSNs were recognized and internalized by CD20 antigen positive Raji cells compared to DMSNs. Furthermore, the prepared RDMSNs exhibited higher cytotoxicity effect and apoptosis efficiency compared to those of Free DOX and DMSNs at the same DOX concentration in the CD20 antigen positive Raji cells. Also, Raji cell apoptosis increased when the concentration of the RDMSNs increased. These should be the covalent conjugations of Rituximab as a ligand which provide active targeting and improved endocytosis of RDMSNs by lymphoma B cells by overexpressing CD20 antigen, enhancing drug efficacy and promoting lymphoma B cells death. In this research in vivo, Free DOX only exhibited a moderate inhibition effect on lymphoma growth compared to that of DMSNs, which was different from the results of cell viability evaluation in vitro. It was attributed to the fact that Free DOX rapidly diffused into all tissues of mice after intravenous administration, leading to potential toxic side effect on healthy tissues, thus only small amount of DOX molecule could reach tumor sites in vivo. On the other hand, Free DOX has shorter half life in vivo, and would be eliminated during the blood circulation and metabolism. Additionally, the enhanced antitumor activity, increased cell apoptosis observed via TUNEL staining assay and reduced systemic toxicity of RDMSNs could probably be involved in the following factors: (1) DOX molecule loaded into the hole of MSNs effectively protected it from quick clearance during blood circulation; (2) Enhanced permeability and retention effect of stealth RDMSNs would passively and actively aggregate the RDMSNs at the tumor site; (3) CD20 antigen-mediated cell specific endocytosis or internalization of the RDMSNs were occurred via CD20 antigen positive Raji cells, improving cell uptake of RDMSNs, and promoting the local chemotherapeutics accumulation. (4) Due to the pH-responsive property, the intracellular DOX release of RDMSNs was improved in the endosomes or lysosomes (pH 4.5–5.5) of tumor cells. (5) The enhanced antitumor efficacy of RDMSNs was occurred through inducing lymphoma cells apoptosis. (6) Rituximab as a molecular targeted drug can induce the antibody-dependent cell-mediated cytotoxicity and complement dependent cytotoxicity. Thus, the synergistic effect of the DOX and Rituximab in the RDMSNs resulted in excellent tumor inhibition effect. (7) DOX is an anthraquinone anticancer agent that is commonly used for the treatment of various types of tumors. DOX can specifically inhibit topoisomeraseIIenzyme activity. However, topoisomeraseIIplays an important role in DNA replication [43]. All these results demonstrated that RDMSNs exhibited both the excellent antitumor effect and the reduced systemic toxicity. Taken together, our study displayed that RDMSNs could serve as an effective and safe platform to improve the therapeutic effect of antitumor drugs and reduce the associated side effects.

## Conclusions

In summary, we have designed and constructed a targeted and DOX-loaded drug delivery system based on MSNs with CD20 antigen-mediated cancer cell uptake and intracellular pH-responsive controlled drug release features. More DOX can be released in an acidic environment (pH 5.0) than in a neutral environment (pH 7.4). The in vitro experiments demonstrated that RDMSNs exhibited targeting accumulation and improved the cytotoxic effects on CD20 positive lymphoma B cells. Furthermore, the in vivo investigations confirmed that RDMSNs could efficiently deliver DOX to tumor cells by pH stimuli, thus inducing cell apoptosis and inhibiting tumor growth, while with minimal toxic side effects. These results suggest that this targeted drug delivery system has the potential for applications in B cell lymphoma therapy.

## Abbreviations

CCK-8: Cell Counting Kit-8
CLSM: Confocal laser scanning microscope
DAPI: 4,6-diamino-2-phenyl indole
DMSNs: Doxorubicin -loaded mesoporous silica nanoparticles
DOX: Doxorubicin
EDC: 1-ethyl-3-(3-dimethylaminopropyl) carbodiimide hydrochloride
FCM: Flow cytometry
FTIR: Fourier transform infrared
H&E: Hematoxylin and eosin
MFI: Mean fluorescence intensity
MSNs: Mesoporous silica nanoparticles
NHS: N-hydroxysuccinimide
PBS: Phosphate buffer saline
PEG: Poly (ethylene glycol)
RDMSNs: Rituximab-conjugated and doxorubicin-loaded mesoporous silica nanoparticles
RMSNs: Rituximab-conjugated mesoporous silica nanoparticles
SEM: Scaning electron microscope
TEM: Transmission electron microscopy
TUNEL: Terminal deoxynucleotidyl transferase dUTP nick end labeling

## References

[1] Habibi-Anbouhi M, Azadmanesh K, Behdani M, Hajizadeh-Saffar E, Vahabpour R, Shokrgozar MA (2015). Development and characterization of a new antipeptide monoclonal antibody directed to human CD20 antigen. Cancer Biother Radiopharm.

[2] Osumi T, Mori T, Fujita N, Saito AM, Nakazawa A, Tsurusawa M, et al. Relapsed/refractory pediatric B-cell non-Hodgkin lymphoma treated with rituximab combination therapy: a report from the Japanese pediatric leukemia/lymphoma study group. Pediatr Blood Cancer. 2016;63:1794–9. 10.1002/pbc.26105 27314926

[3] Zhou D, Li L, Bao C, Zhu J, Zhu L, Yang X (2015). Replacement of conventional doxorubicin by pegylated liposomal doxorubicin in standard RCHOP chemotherapy for elderly diffuse large B-Cell lymphoma: a retrospective study in China. Int J Clin Exp Med.

[4] Tang F, Li L, Chen D (2012). Mesoporous silica nanoparticles: synthesis, biocompatibility and drug delivery. Adv Mater (Deerfield Beach, Fla).

[5] Ashley CE, Carnes EC, Phillips GK, Padilla D, Durfee PN, Brown PA (2011). The targeted delivery of multicomponent cargos to cancer cells by nanoporous particle-supported lipid bilayers. Nat Mater.

[6] Yang P, Gai S, Lin J (2012). Functionalized mesoporous silica materials for controlled drug delivery. Chem Soc Rev.

[7] Wu SH, Mou CY, Lin HP (2013). Synthesis of mesoporous silica nanoparticles. Chem Soc Rev.

[8] Li J, Liu F, Shao Q, Min Y, Costa M, Yeow EK (2014). Enzyme-responsive cell-penetrating peptide conjugated mesoporous silica quantum dot nanocarriers for controlled release of nucleus-targeted drug molecules and real-time intracellular fluorescence imaging of tumor cells. Adv Healthc Mater.

[9] Li J, Wu S, Wu C, Qiu L, Zhu G, Cui C (2016). Versatile surface engineering of porous nanomaterials with bioinspired polyphenol coatings for targeted and controlled drug delivery. Nanoscale.

[10] Yu Z, Li N, Zheng P, Pan W, Tang B (2014). Temperature-responsive DNA-gated nanocarriers for intracellular controlled release. Chem Commun (Cambridge, England).

[11] Aznar E, Mondragon L, Ros-Lis JV, Sancenon F, Marcos MD, Martinez-Manez R (2011). Finely tuned temperature-controlled cargo release using paraffin-capped mesoporous silica nanoparticles. Angew Chem Int Ed Eng.

[12] Fedoryshin LL, Tavares AJ, Petryayeva E, Doughan S, Krull UJ (2014). Near-infrared-triggered anticancer drug release from upconverting nanoparticles. ACS Appl Mater Interfaces.

[13] Li M, Yan H, Teh C, Korzh V, Zhao Y (2014). NIR-triggered drug release from switchable rotaxane-functionalized silica-covered au nanorods. Chem Commun (Cambridge, England).

[14] Zhang B, Luo Z, Liu J, Ding X, Li J, Cai K (2014). Cytochrome c end-capped mesoporous silica nanoparticles as redox-responsive drug delivery vehicles for liver tumor-targeted triplex therapy in vitro and in vivo. J Control Release.

[15] Dai L, Li J, Zhang B, Liu J, Luo Z, Cai K (2014). Redox-responsive nanocarrier based on heparin end-capped mesoporous silica nanoparticles for targeted tumor therapy in vitro and in vivo. Langmuir ACS J Surfaces Colloids.

[16] Luo Z, Ding X, Hu Y, Wu S, Xiang Y, Zeng Y (2013). Engineering a hollow nanocontainer platform with multifunctional molecular machines for tumor-targeted therapy in vitro and in vivo. ACS Nano.

[17] Zhu L, Wang T, Perche F, Taigind A, Torchilin VP (2013). Enhanced anticancer activity of nanopreparation containing an MMP2-sensitive PEG-drug conjugate and cell-penetrating moiety. Proc Natl Acad Sci U S A.

[18] Zhang G, Yang M, Cai D, Zheng K, Zhang X, Wu L (2014). Composite of functional mesoporous silica and DNA: an enzyme-responsive controlled release drug carrier system. ACS Appl Mater Interfaces.

[19] Tan L, Yang MY, Wu HX, Tang ZW, Xiao JY, Liu CJ (2015). Glucose- and pH-responsive nanogated ensemble based on polymeric network capped mesoporous silica. ACS Appl Mater Interfaces.

[20] Muhammad F, Wang A, Guo M, Zhao J, Qi W, Yingjie G (2013). pH dictates the release of hydrophobic drug cocktail from mesoporous nanoarchitecture. ACS Appl Mater Interfaces.

[21] Li LL, Xie M, Wang J, Li X, Wang C, Yuan Q (2013). A vitamin-responsive mesoporous nanocarrier with DNA aptamer-mediated cell targeting. Chem Commun (Cambridge, England).

[22] Zhang P, Cheng F, Zhou R, Cao J, Li J, Burda C (2014). DNA-hybrid-gated multifunctional mesoporous silica nanocarriers for dual-targeted and microRNA-responsive controlled drug delivery. Angew Chem Int Ed Eng.

[23] Meng H, Xue M, Xia T, Zhao YL, Tamanoi F, Stoddart JF (2010). Autonomous in vitro anticancer drug release from mesoporous silica nanoparticles by pH-sensitive nanovalves. J Am Chem Soc.

[24] Dai L, Zhang Q, Li J, Shen X, Mu C, Cai K (2015). Dendrimerlike mesoporous silica nanoparticles as pH-responsive nanocontainers for targeted drug delivery and bioimaging. ACS Appl Mater Interfaces.

[25] He X, Alves CS, Oliveira N, Rodrigues J, Zhu J, Banyai I (2015). RGD peptide-modified multifunctional dendrimer platform for drug encapsulation and targeted inhibition of cancer cells. Colloids Surf B Biointerfaces.

[26] Zhang C, Wang W, Liu T, Wu Y, Guo H, Wang P (2012). Doxorubicin-loaded glycyrrhetinic acid-modified alginate nanoparticles for liver tumor chemotherapy. Biomaterials.

[27] Zhao Q, Geng H, Wang Y, Gao Y, Huang J, Wang Y (2014). Hyaluronic acid oligosaccharide modified redox-responsive mesoporous silica nanoparticles for targeted drug delivery. ACS Appl Mater Interfaces.

[28] Brevet D, Gary-Bobo M, Raehm L, Richeter S, Hocine O, Amro K, et al. Mannose-targeted mesoporous silica nanoparticles for photodynamic therapy. Chem Commun (Cambridge, England). 2009;28:1475–7. 10.1039/b900427k 19277361

[29] Fang IJ, Slowing II, Wu KC, Lin VS, Trewyn BG (2012). Ligand conformation dictates membrane and endosomal trafficking of arginine-glycine-aspartate (RGD)-functionalized mesoporous silica nanoparticles. Chemistry (Weinheim an der Bergstrasse, Germany).

[30] Yang X, Liu X, Liu Z, Pu F, Ren J, Qu X (2012). Near-infrared light-triggered, targeted drug delivery to cancer cells by aptamer gated nanovehicles. Adv Mater (Deerfield Beach, Fla).

[31] Luo Z, Cai K, Hu Y, Zhao L, Liu P, Duan L (2011). Mesoporous silica nanoparticles end-capped with collagen: redox-responsive nanoreservoirs for targeted drug delivery. Angew Chem Int Ed Engl.

[32] Zhou S, Zheng S, Shan Y, Li L, Zhang X, Wang C (2016). Rituximab-conjugated and doxorubicin-loaded microbubbles combined with ultrasound irradiation inhibits proliferation and induces apoptosis in Raji cell lines. Oncol Rep.

[33] Song ZL, Chen HL, Wang YH, Goto M, Gao WJ, Cheng PL (2015). Design and synthesis of novel PEG-conjugated 20 (S)-camptothecin sulfonylamidine derivatives with potent in vitro antitumor activity via cu-catalyzed three-component reaction. Bioorg Med Chem Lett.

[34] Christie RJ, Matsumoto Y, Miyata K, Nomoto T, Fukushima S, Osada K (2012). Targeted polymeric micelles for siRNA treatment of experimental cancer by intravenous injection. ACS Nano.

[35] Xie M, Shi H, Li Z, Shen H, Ma K, Li B (2013). A multifunctional mesoporous silica nanocomposite for targeted delivery, controlled release of doxorubicin and bioimaging. Colloids Surf B Biointerfaces.

[36] Dai B, Hu Y, Duan J, Yang XD (2016). Aptamer-guided DNA tetrahedron as a novel targeted drug delivery system for MUC1-expressing breast cancer cells in vitro. Oncotarget.

[37] Hayward SL, Wilson CL, Kidambi S (2016). Hyaluronic acid-conjugated liposome nanoparticles for targeted delivery to CD44 overexpressing glioblastoma cells. Oncotarget.

[38] Zheng H, Tai CW, Su J, Zou X, Gao F (2015). Ultra-small mesoporous silica nanoparticles as efficient carriers for pH responsive releases of anti-cancer drugs. Dalton Trans.

[39] Xie X, Li F, Zhang H, Lu Y, Lian S, Lin H (2016). EpCAM aptamer-functionalized mesoporous silica nanoparticles for efficient colon cancer cell-targeted drug delivery. Eur J Pharm Sci.

[40] Zhao Q, Liu J, Zhu W, Sun C, Di D, Zhang Y (2015). Dual-stimuli responsive hyaluronic acid-conjugated mesoporous silica for targeted delivery to CD44-overexpressing cancer cells. Acta Biomater.

[41] She X, Chen L, Velleman L, Li C, Zhu H, He C (2015). Fabrication of high specificity hollow mesoporous silica nanoparticles assisted by Eudragit for targeted drug delivery. J Colloid Interface Sci.

[42] Liu J, Zhang B, Luo Z, Ding X, Li J, Dai L (2015). Enzyme responsive mesoporous silica nanoparticles for targeted tumor therapy in vitro and in vivo. Nanoscale.

[43] Wang H, Agarwal P, Zhao S, Xu RX, Yu J, Lu X (2015). Hyaluronic acid-decorated dual responsive nanoparticles of Pluronic F127, PLGA, and chitosan for targeted co-delivery of doxorubicin and irinotecan to eliminate cancer stem-like cells. Biomaterials.

